# Allostatic load modelling, lifestyle and cardiological risk factor: evidence for integrating patient profiling in the optimisation of pharmacological therapies during follow-ups in hospital setting – PLAY-UP cohort study protocol

**DOI:** 10.1136/bmjopen-2023-082459

**Published:** 2024-11-20

**Authors:** Dina Di Giacomo, Luigi Sciarra, Liuba Fusco, Antonio Gianluca Robles, Andrej Pernat, Silvio Romano

**Affiliations:** 1 Life, Health and Environmental Sciences Department, University of L'Aquila, L'Aquila, Italy; 2 Di Lorenzo Clinic, Avezzano, Italy; 3 Cardiology Department, Chelsea and Westminster Hospital, London, UK; 4 Cardiology Hospital, L. Bonomi Hospital, Bari, Italy; 5 Department of Cardiology, University Medical Centre Ljubljana, Ljubljana, Slovenia; 6 Heart Failure Clinic, ASL 1 Avezzano Sulmona L'Aquila, L'Aquila, Italy

**Keywords:** Cardiovascular Disease, Hypertension, Anxiety disorders

## Abstract

**Abstract:**

**Introduction:**

The allostatic load (AL) is a framework for conceptualising the physiological multisystemic impact of prolonged exposure to stress and its related side effects on mental health.

Stress due to AL can influence the development and outcomes of cardiovascular diseases. AL increases the risk of coronary and peripherical artery diseases. AL emerges from the detection of emotional dimensions related to the disease, low psychosocial functioning and high rates of psychopathological signs in patients with hypertension or coronary heart disease.

**Method and analysis:**

The primary endpoint of the PLAY-UP protocol is the implementation of a multidimensional model underlying the clinical treatment of patients with cardiovascular disease through the integration of medical and psychological clinical variables.

PLAY-UP is a cohort study that will last for 24 months. 200 participants will be recruited and divided into three groups: early disease, midterm disease and long disease. All patients will undergo a clinical evaluation based on the detection of biological, medical and psychological indicators and variables. The evaluation battery will comprise three types of measurements: medical, psychological and pharmacological treatments. Clinical and psychological measurements will be processed in an integrated manner through the combination of all variables examined, elaborating the Allostatic Load Index from a longitudinal time perspective. The Allostatic Load Index will be calculated by measuring the z-score.

**Ethics and dissemination:**

Ethical Committee Approval was obtained from CEtRA Abruzzo Region (IT) (ID 0461499/23). The results of the present project will be published in peer-reviewed journals, disseminated electronically and in print, and presented as abstracts and/or personal communications during national and international conferences.

**Strengths and limitations of this study**

- Multidimensional model by integrating medical and psychological clinical variables.
- Medical/psychological measurements and pharmacological treatments will be analysed into patient profile.
- The integration of clinical and psychological measurements will be processed elaborating the Allostatic Load Index.
- Unbalanced distribution into three patient groups based on disease timing.

## Introduction

 The allostatic load (AL) is a framework for conceptualising the physiological multisystemic impact of prolonged exposure to stress and its related side effects on mental health. McEwan introduced AL regarding long exposure to chronic psychosocial and physical challenges in individuals reacting to high stress levels and fluctuating neural and neuroendocrine responses.^1^ It is derived from the definition of allostasis, which is an organism’s ability to achieve stability by changing,^2^ and from the fact that healthy functioning requires continuous physiological internal adaptation.^3^ In brief, AL^2^,^4^ reflects the cumulative effect of daily life experiences involving routine events (imperceptible and long-standing life situations) as well as major challenges (life events) and also includes the physiological consequences resulting from the implementation of behaviours harmful to one’s health (eg, poor sleep, lack of physical exercise, smoking, alcohol consumption and an unhealthy diet). When the demands of the context overwhelm the individual’s ability to cope with them, allostatic overload ensues^5 6^ that is, a transition occurs towards an extreme state in which stress response systems are repeatedly activated, and buffers are inadequate.^7^ The situations that can lead to the development of allostatic load/overload are as follows: (a) chronic stress: exposure to frequent stressors that can determine a state of chronic stress and repeated physiological arousal; (b) failure to adapt: lack of adaptation to repeated stressors; (c) response blocking failure: inability to stop the stress response after the stressor is no longer present; and (d) inadequate hormonal response: insufficient allostatic response for coping with the stressor.

Several studies have focused on identifying allostatic load using biological markers. Seemann *et al*^8^ identified 10 biological parameters: (1) cortisol, (2) dehydroepiandrosterone, (3) epinephrine, (4) norepinephrine, (5) cholesterol, (6) glycosylated haemoglobin, (7) systolic and diastolic blood pressure, (8) Body Mass Index (BMI) and (9) waist-hip ratio. The first four parameters were considered primary mediators of the allostatic load because of their immediate correlation with adrenal function; whereas, the remaining parameters were defined as secondary mediators.

Additional biomarkers (glucose levels, lipid profiles, interleukin-6 and heart rate variability) were subsequently recognised as being attributable to AL and included in the cumulative AL Index (Allostatic Load Index, ALI).^8^,^10^ Several studies have highlighted that this index is the best predictor of mortality and decline in physical functioning^11^ compared with individual biomarkers.^912^,^14^

A biological perspective must be complemented by psychological measurements that allow for building a comprehensive understanding of allostatic load, overload and related clinical phenomena. For the psychological measurement of allostatic load, the following clinical criteria were applied (progression from A to B):

Criterion A: Presence of an identifiable source of distress in the form of recent life events and/or chronic stress: The stressor is considered to challenge or exceed an individual’s coping abilities when its full nature and characteristics are assessed.

Criterion B: The stressor is associated with one or more of the following characteristics within 6 months of its onset:

1. At least two of the following symptoms must be observed in daily life: difficulty falling asleep, restless sleep, difficulty waking up in the morning, lack of energy, dizziness, generalised anxiety, irritability, sadness and demoralisation.
2. Significant impairment in social or occupational functioning.
3. Significant impairment in contextual mastery (feeling overwhelmed by the demands of daily life).

### Stress and cardiovascular disease

Stress due to AL can influence the development and outcomes of cardiovascular disease (CVD). AL increases the risk of coronary heart disease,^13^ ischaemic heart disease^14^ and peripheral arterial disease.^15^,^17^

AL emerges from the detection of emotional dimensions related to the disease, low psychosocial function and high rates of psychopathological signs in patients with hypertension and coronary heart disease.^18^,^20^ Similarly, AL seems to be related to an increase in psychological distress, depression and anxiety in patients with atrial fibrillation.^21^ The study focused on patients who were exposed to clinical intervention for an implantable cardioverter defibrillator^22^ and found that, for 16.2%, AL were moderate; whereas, 4.3% showed high AL. In particular, one study suggested that the presence of AL before implantation was the only significant predictor of subsequent adverse cardiac outcomes, including complications and death after implantation; this was significantly associated with hyperglycaemia among cardiac risk factors.^23^

Among patients with hypertension, 32.5% showed AL, with significantly higher levels of psychological distress and a higher prevalence of psychosomatic syndromes. The presence of AL in patients with hypertension has been associated with lower levels of well-being and quality of life.^24^

To date, studies have focused primarily on the detection of AL in cardiovascular pathologies by identifying patient adaptive processes. The influence of AL has assumed clinical relevance in the management of cardiovascular health through patient profiling, and the identification of its strengths and weaknesses could contribute to the optimisation of pharmacological therapy for chronic diseases.

Using a biopsychosocial model,^25^ the holistic framework of multidimensionality is based on the integration of different domains that make up the construct of quality of life in patient care. The PLAY-UP protocol aims to investigate the interaction between risk and protective factors in the clinical treatment of patients with cardiovascular risk and/or cardiac pathology according to the multidimensional approach of the complex dynamics of the AL framework.^26^

### Objectives

The primary endpoint of the PLAY-UP protocol is the implementation of a multidimensional model underlying the clinical treatment of patients with cardiovascular disease by integrating medical and psychological clinical variables. In particular, the PLAY-UP protocol aims to optimise pharmacological treatments by enhancing patients’ empowerment in the management of their health by modulating the determinants of AL.

The secondary endpoints are as follows:

- Identification of the clinical dimensional factors of cardiac patients undergoing pharmacological treatment in both postsurgical and nonsurgical conditions. The aim is to detect the processes of change in clinical conditions depending on the evaluation and modulation of AL.
- Detecting predictive patterns for managing and monitoring clinical variables in cardiology after considering changes in pharmacological treatment and quality of life variables in prolonged care, delineating patient profiling from a longitudinal perspective.
- To analyse the influence of emotional and physiological triggers on adherence to therapy, we aim to investigate the impact of affective (emotional triggers) and organic (physical triggers) factors on the development of clinical pathways.

Our hypothesis is that the medical aspects of CVD disease, related clinical common features and patient’s adherence to therapies are influenced by the psychological profile: they can blunt, overemphasise as well as fit in them.

## Methods

### Study setting

The study will be conducted as part of the ‘Corporate programme for hospital-territory integration and optimisation of diagnostic-therapeutic pathways in the management of heart failure’ of ASL 1 Abruzzo, IT (Head Professor: S. Romano), at the Cardiovascular Diagnostics and Arrhythmology Division of the ‘Di Lorenzo’ Nursing Home in Avezzano, IT (Head Professor: Luigi Sciarra).

### Study sample

The population that will participate in the study consists of 200 patients with cardiovascular disease in the 18–80 age range; they are undergoing clinical treatment.

Patients affected by the following chronic cardiovascular diseases will be enrolled (inclusion criteria):

- Heart failure will be diagnosed according to the criteria of the European Guidelines.^26^
- Ischaemic heart disease (a history of myocardial infarction, coronary revascularisation through angioplasty or bypass).
- Cardiomyopathy of any aetiology (dilated, hypertrophic, arrhythmogenic or restrictive).
- Patients with valvular disease undergoing surgical or interventional treatment.

The exclusion criteria will be as follows:

- Presence of oncological pathology in the disease activity phase (ie, oncological follow-up).
- Undergoing pharmacological treatment for psychiatric and/or neurological pathologies.

All patients eligible for the PLAY-UP protocol will have to sign an informed consent form for enrolment.

### Measures

**Figure 1:**
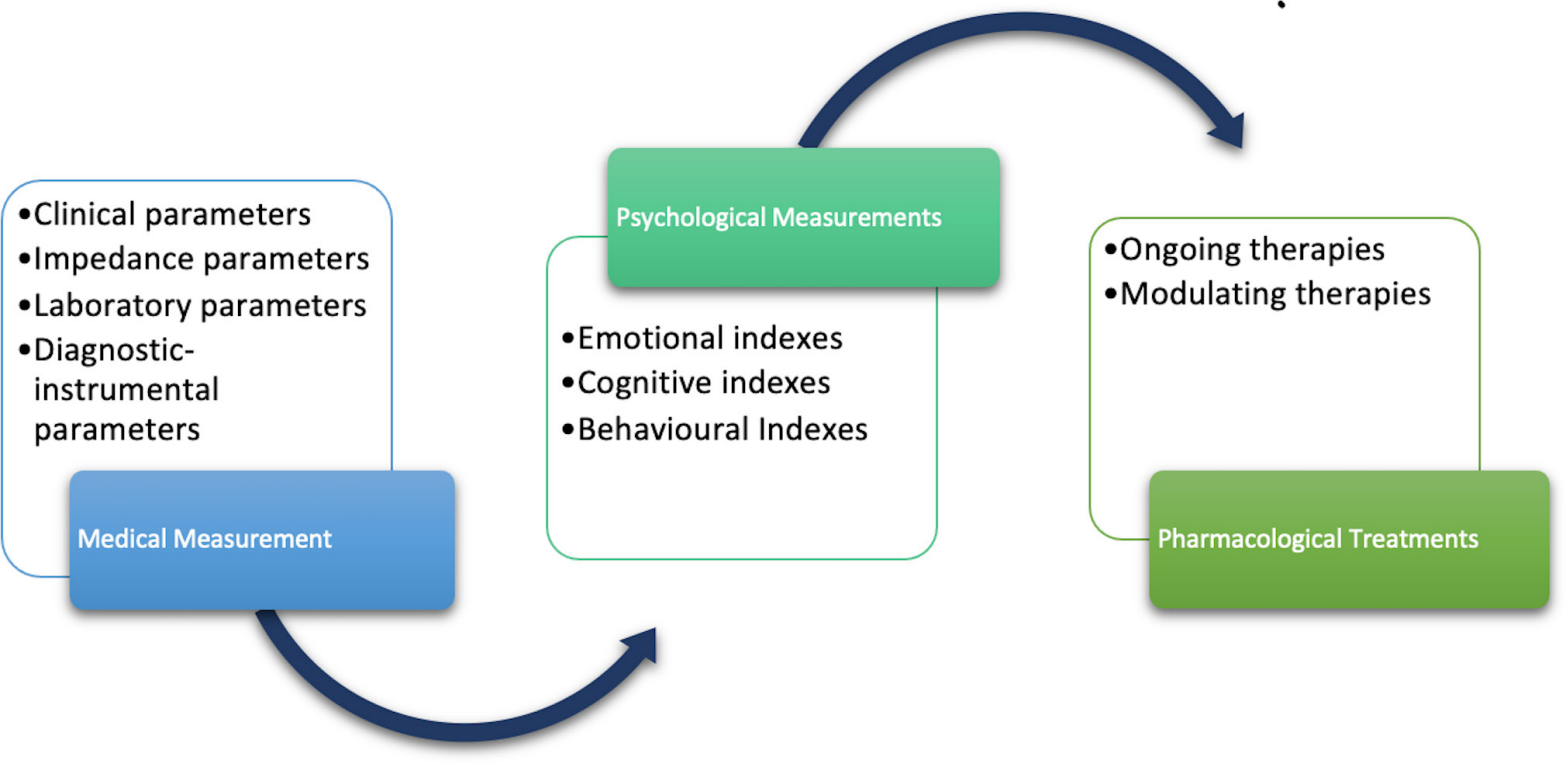
Applied measurement in the PLAY-UP project.

All patients will undergo a clinical evaluation based on the detection of biological, medical and psychological indicators and variables. The evaluation battery comprises three types of measurements: medical, psychological and pharmacological treatments (see figure 1).

### Medical measurements

#### Clinical parameters

Clinical measurements will be obtained from the pre-established clinical health protocol as part of the prescribed checks (follow-ups) in clinical practice by medical staff.

The following clinical indicators, which are part of the parameters evaluated in usual clinical practice in patients with pathologies identified according to the inclusion criteria, will be collected.

- Subjective symptoms (New York Heart Association – NYHA – classification).
- Sloping oedema.
- Oxygen saturation by pulse oximetry.
- Diastolic blood pressure: Three consecutive and sequential measurements of the average value.
- Evaluation of systolic blood pressure: Three consecutive and sequential measurements of the average value.
- Heart rate.

#### Impedance parameters

- Basal metabolic rate: This is expressed in kilocalories (kcal) and is the amount of daily energy necessary to maintain vital functions.
- Qualitative and quantitative body analysis includes fat mass value, body fat, muscle mass, body weight without fat, ideal body weight, muscle frequency, body weight without fat, body type, bone mass, visceral fat, subcutaneous fat, proteins, water body weight, protein mass, % body water, % skeletal muscle and water weight.
- BMI is calculated based on the relationship between weight and height.

#### Laboratory parameters

- Low-density lipoprotein cholesterol.
- Brain natriuretic peptide and N-terminal pro-b-type natriuretic peptide proteins.

#### Diagnostic-instrumental parameters

- ECG: evaluation of rhythm and heart rate, measurement of the duration of the QRS complex and corrected PR and QT intervals, and presence of ventricular and/or supraventricular extrasystoles.
- Echocardiogram (on clinical indication): evaluation of biventricular systolic and diastolic function indices through two-dimensional structural evaluation, colour Doppler, analysis of the transmitral and transtricuspid flow profile, pulmonary venous flow and tissue Doppler of the mitral annulus.
- Holter ECG (on clinical indication): evaluation of rhythm and heart rate (minimum, average and maximum), quantification of ventricular and/or supraventricular extrasystoles, and evaluation of the variability of the RR interval.

### Psychological measurements

Subsequently, a standardised psychological battery composed of 9 psychological tests divided into emotional, cognitive and behavioural indexes will be applied; Italian translation and adaptation of them will be used.

#### Emotional indexes

- Depression, Anxiety, Stress Scale (DASS-21).^27^ The DASS-21 is a self-report that measures the degree of severity of the core symptoms for emotional dimensions rather than a categorical conception of a psychological disorder. It is composed of 21 questions with responses on a 4-point Likert-type scale, and it measures 3 sets of self-report scales designed to measure the emotional states of depression, anxiety and stress. The inventory demonstrated good reliability (α=0.90).
- General Self-Efficacy (GSE).^28^ It is an anxiety-screening test that assesses the severity of generalised anxiety disorders. This scale measures self-efficacy and is made up of 10 items. GSE is correlated to emotion, optimism and work satisfaction. Negative coefficients were found for depression, stress, health complaints, burnout and anxiety. The total score is calculated by finding the sum of the all items. Internal reliability for GSE is Cronbach’s alphas between 0.76 and 0.90.
- Barratt Impulsiveness Scale (BIS-11).^29^ The BIS-11 is a self-report measure of impulsive personality traits and identifies three factors that express three different constructs of impulsivity: (1) cognitive impulsivity, which is understood as inattention and cognitive instability; (2) motor impulsivity, which is understood as motor instability and a lack of perseverance; and (3) non-planning impulsivity, which is understood as a lack of self-control and intolerance to cognitive complexity. The internal consistency coefficients for the BIS-11 are on total score that ranges from 0.79 to 0.83.
- Cardiac Anxiety Questionnaire (CAQ).^30^ The CAQ measures heart-focused anxiety, as this may be a more specific form of anxiety relevant to heart patients. It consists of 18 items, and it can be divided in three subscales (Fear, 8 items; Avoidance, 5 items; Attention, 5 items). Each item is rated on a 5-point Likert scale with scores ranging from 0 (never) to 4 (always). A high score indicates a greater number of symptoms, greater frequency or both. We will apply the experimental version of Italian adaptation provided by the ‘La Cattolica Research Group’.

#### Cognitive indexes

- Mini Mental State Examination (MMSE).^31^ This is a neuropsychological test used for evaluating intellectual efficiency disorders and cognitive deterioration. The MMSE is often used as a screening tool used by to check for cognitive impairment to investigate subjects with dementia and neuropsychological syndromes of different natures. It is composed of 11 items. The top score for the MMSE is 30, and the score of 25 or higher is said to be normal.
- Cognitive Reserve Index questionnaire (CRI-q).^32^ CRI-q was constructed based on the main cognitive reserve indices reported in the literature. The test is divided into three indices: CRI-School, CRI-Work and CRI-FreeTime; subsequently, these are combined into a single value called the ‘Cognitive Reserve Index’ (CRI). The final score of the CRI-q questionnaire and its three sub-indexes are expressed on a scale with a mean of 100 and an SD of 15 (exactly like IQ).

#### Behavioural indexes

- Self-Care of Chronic Illness Inventory (SELF-CARE).^33^ The instrument has been developed to measure self-care behaviours in people with all types and numbers of chronic diseases. It currently has four dimensions: (1) Self-Care Maintenance—that is, the patient’s ability to implement self-care behaviours to preserve the stability of the chronic disease and prevent complications; (2) Self-Care Monitoring—that is, the patients’ ability to monitor and recognise early specific signs and symptoms of their chronic disease as well as predictors of complications; (3) Self-Care Management—that is, the patient’s ability to manage signs, symptoms and the complications associated with them; and (4) Patient Self-Care Confidence—that is, the level of self-confidence in carrying out specific self-care activities.
- WHO Quality of Life Brief Version (WHOQOL-BREF).^34^ It is a self-report questionnaire which assesses four domains of quality of life (QOL): physical health, psychological health, social relationships and environment. In addition, there are two items that measure overall QOL and general health. The instrument is composed of 26 items that investigate four areas that represent the construct of quality of life: (1) physical health, (2) psychological health, (3) social relations and (4) environment. The four domain scores are scaled in a positive direction with higher scores indicating a higher quality of life.
- EQ-5D (EUROQOL).^35^ The EQ-5D is a standardised measure of health-related quality of life developed by the EuroQoL Group to provide a simple and generic questionnaire for use in clinical and economic evaluations and population health surveys. The test is composed of five items and one for each five dimensions as (a) mobility, (b) self-care, (c) usual activities, (d) pain/discomfort, (e) and anxiety/depression.

### Procedures

Trained medical, psychological and nursing researchers will assess physical and mental functioning parameters on enrolment in the study (baseline visit) and, with respect to the baseline visit, on 6 and 12 and at 18 months. Assessments will be performed in ambulatory care settings at both study sites. See below paragraph ‘Study Design’ for recruiting procedure.

### Pharmacological treatments based on clinical protocol

Ongoing pharmacological treatments and modulation of therapies at follow-up will be detected according to the indications of specific guidelines for individual pathologies and the rules of Good Clinical Practice.^36^

### Integration of clinical and psychological measurements: allostatic load index

Clinical and psychological measurements will be processed in an integrated manner through the combination of all variables examined, elaborating the Allostatic Load Index from a longitudinal time perspective. The Allostatic Load Index (ALI) will be calculated by measuring the z-scores and identifying the number of clinical and psychological values that fall within a high-risk percentile (ie, lower or upper quartile) based on the normal distribution of the values used in clinical practice.^37^ Through integrated data processing, the strengths and weaknesses of drug therapy and lifestyle management will be identified. Through the identification of integrated clinical and psychological variables, a patient profile will be obtained, which will determine the modelling of pharmacological therapy aimed at reducing the cumulative effects of the management of complex physical symptoms and the related psychological implications in terms of the patient’s quality of life.

### Study design

The PLAY-UP project is a cohort study that will last 24 months. Participants will be recruited and divided into three groups: early disease (ED), mid-term disease (MD) and long-term disease (LD). Eligible patients are subjects diagnosed with cardiovascular disease as indicated in above ‘Study Sample’ section. The criterion for subdivision into the three groups will be the time elapsed since the diagnosis of a pathology with the indicated characteristics (heart failure, ischaemic heart disease, cardiomyopathy, valvular disease subjected to surgical/interventional treatment). The ED group will include patients with an elapsed time period of less than 2 years since diagnosis; the MD group will include patients with an elapsed time period of 2–4 years from diagnosis; and the LD group will include patients with an elapsed diagnosis time period exceeding 4 years.

The clinical and psychological findings provided for this protocol will be conducted during follow-up evaluations used in usual clinical practice.

**Figure 2:**
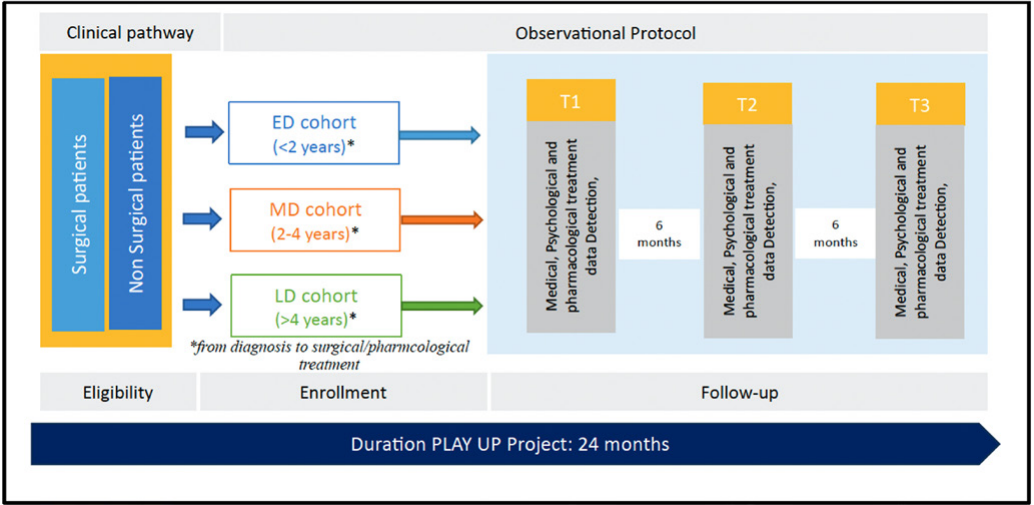
PLAY-UP study design.

Figure 2 presents a representation of the study design.

### Statistical analysis

The study data will be incorporated into a database and statistically processed by blinded procedure.

First, qualitative data analysis will be conducted by using interpretive descriptions to generate areas and subareas and by deductively using framework analysis^38^ to map the data.

The quantitative data will be analysed. Descriptive, linear regression and correlation analyses will be conducted with Jamovi (significance level α<0.5).

Path model analysis will be performed to evaluate the effects of a set of psychological and quality of life management variables that act on the management of health among CVD patients through multiple causal paths, and linear regression will be conducted to detect the relationship between clinical, psychological, quality of life and pharmacological variables.

### Data monitoring and safety

All participants will be monitored by both research staff and clinical teams based on standard hospital care and protocols. The research team will monitor patients for adverse events. Data monitoring will include periodic reviews of recruitment progress, retention, serious adverse events and general study progress.

### Current status

The PLAY-UP project will begin enrolling patients in January 2024. To date, two medical centres in Italy will be recruiting patients. Recruitment is expected to be completed by December 2025.

### Patient and public involvement

The patients and/or the public will not be involved in the design, conduct, reporting or dissemination plans of this research.

### Access to data

The research data may be shared after de-identification on reasonable request. Proposals to access the data will be reviewed and approved by the Principal Investigator of the PLAY-UP trial (Professor Silvio Romano, silvio.romano@univaq.it).

### Data management

The participants’ paper and electronic files will be maintained in secure storage on a password-protected computer throughout the project. Data will then be stored at the University of L’Aquila (IT) for at least 5 years after publication, after which these will be destroyed according to university protocols. Non-numerical data will be coded according to predefined coding definitions. Data entry screening will be conducted at the time of data entry to ensure that there are no inconsistencies between the paper-based and electronic data.

## Ethics and dissemination

Ethical Committee Approval was obtained from CEtRA Abruzzo Region (IT) (ID 0461499/23). The results of the present project will be published in peer-reviewed journals, disseminated electronically and in print, and presented as abstracts and/or personal communications during national and international conferences. Written informed consent will be treated as a mandatory component of this research according to the guidelines of the Declaration of Helsinki.^39^ National and international regulations on patient privacy will be followed.
