# Supplementary material for: Allostatic load modelling, lifestyle and cardiological risk factor: evidence for integrating patient profiling in the optimisation of pharmacological therapies during follow-ups in hospital setting – PLAY-UP cohort study protocol
Source: BMJ Open. 2024 Nov 20;14(11):e082459. doi: 10.1136/bmjopen-2023-082459 (PMC11580299; doi:10.1136/bmjopen-2023-082459)
Supplement: Supplementary file 1 [file bmjopen-2023-082459.reviewer_comments.pdf]

PEER REVIEW HISTORY

BMJ Open publishes all reviews undertaken for accepted manuscripts. Reviewers are asked to complete a checklist review form and are provided with free text boxes to elaborate on their assessment. These free text comments are reproduced below.

ARTICLE DETAILS

Title (Provisional)

Allostatic load modelling, lifestyle, and cardiological risk factor: evidence for integrating patient profiling in the optimisation of pharmacological therapies during follow-ups in hospital setting. PLAY-UP cohort-study protocol

Authors

Di Giacomo, Dina; Sciarra, Luigi; Fusco , Liuba; Robles,, Antonio Gianluca; Pernat, A; Romano, Silvio

---

VERSION 1 - REVIEW

**Reviewer**	**1**
**Name**	**Pietrabissa, Giada**
**Affiliation**	**Università Cattolica del Sacro Cuore, Department of Psychology**
**Date**	**09-Jan-2024**
**COI**	**none**

This very interesting contribution describes the implementation of a multidimensional model aimed at increasing patients' self-management by modulating the determinants of allostatic load. The paper is of good quality, and only a few suggestions are provided below:

p.4 – line 5: Please delete the term 'background'

p. 7 – line 48-49: Please specify the study design (instead stated at p.12 line 11-12) in the designated section

p.7 – line 60: study sample or study population. Also, please provide the sample size calculation.

Measure: I recommend the authors consistently use bullet points and provide a more comprehensive description of the measures of interest, particularly emotional, cognitive and behavioral indexes

The reference for the Italian translation and validation of the questionnaire should also be provided.

Is it not clear why the authors listed the measures of quality of life as behavioral rather than psychological

p. 8 – line 38 Please specify the acronyms LDL (low-density lipoprotein)

p.11 – line 27-28: I suggest the authors to remove the term ‘outcomes’ and to clarify the follow-up points

Please clearly describe the recruitment and screening process.

p.12 – line 10: it is suggested to replace the subtitle ‘study design’ with ‘procedures’

It is recommended to add sections: ‘expected results’ and ‘conclusions’ at the end of the manuscript

p.13 – line 10: please replace the word ‘CDV’ with ‘CVD’

In addition:

I recommend the authors specify acronyms only once in the text and use them consistently. For example: cardiovascular disease (CVD)

I suggest the authors standardize verbal time to the future throughout the text. I also recommend to uniform line spacing throughout the manuscript

---

Reviewer 2

Name Sampaio, Karla

Affiliation Postgraduate Program in Pharmaceutical Sciences, Pharmaceutical Sciences

Date 14-Jun-2024

COI None

The study protocol submitted by Giacomo and colleagues to the BMJ intends to implement a multidimensional model treatment for patients with cardiovascular disease based on the allostatic load index (ALI). The study is interesting and worthy investigating. However, some points need clarification.

Introduction

The background is well-anchored on literature evidence. However, considering the endpoints, what do the authors expect to find by the end of the study? What is their hypothesis?

Objectives

The specific aims towards the secondary endpoints seem too broad. Can the authors be more specific regarding it?

Methods

1. How did the authors define the sample size? Please present the statistical parameters used to define it and the supporting reference.

2. In the inclusion criteria, the authors state that they will enroll Heart failure patients. However, most evidence presented in the introduction, regarding the relationship between allostatic load and cardiovascular disease, is among hypertensive patients. If the idea is to develop a preventive model based on the AL Index, why not include hypertensive patients before heart failure dysfunction is installed?

3. Explain why there is no control arm in the study design.

4. Explain the rationale for excluding patients undergoing pharmacological treatment for psychiatric disorders, once a great part of AL is dependent on psychological factors. Can this introduce a bias in the study?

5. How will the authors control patients' pharmacological treatment for other conditions straightly related to CVD such as diabetes?

6. Justify the inclusion of only two laboratory parameters, once in the introduction, other biochemical markers are used to compose the ALI.

7. In the outcomes section, the authors describe a study sequence of visits at baseline, day 6, 12, and 18 months. However, in figure 2, another timeline interval is stated. Please justify the different intervals and present evidence for supporting the choice of time interval and duration of the study.

8. It isn't clear what will be measured by the end of the study. What are the patients' outcomes to be analyzed?

9. Will statistical analysis be blinded conducted?

10. Provided a more detailed description of statistical analysis, providing supporting evidence.

VERSION 1 - AUTHOR RESPONSE

Reviewer: 1 (R1)
Dr. Giada Pietrabissa, Università Cattolica del Sacro Cuore

R1: Comments to the Author:
This very interesting contribution describes the implementation of a

multidimensional model aimed at increasing patients' self-management by modulating the determinants of allostatic load.

Authors: Thanks for the appreciation. We will go on following your suggestions.

R1: The paper is of good quality, and only a few suggestions are provided below:

p.4 – line 5: Please delete the term 'background'

Authors: Done!

R1: p. 7 – line 48-49: Please specify the study design (instead stated at p.12 line 11-12) in the designated section

Authors: We tried to make the changes, but the paragraph resulted not readable. Please, could you accept the distinguishing between study setting and then study design in order to make clearer the research protocol. We are grateful for that.

R1: p.7 – line 60: study sample or study population. Also, please provide the sample size calculation.

Authors:

1) We specified the 'Study sample' as title of Method subparagraph (Please see pag. 7)

2) We elaborated the sample size as reported in the Protocol on the basis of the theoretical framework discussed in the Introduction paragraph, but we didn't execute specific calculation. Our Protocol study is an observational study based on clinical population and further intervention protocol study will foresee the sample size calculation.

R1: Measure: I recommend the authors consistently use bullet points and provide a more comprehensive description of the measures of interest, particularly emotional, cognitive and behavioral indexes

Authors: As suggested, we worked both suggestions:

a) bullet points have been provided for all applied exam/tests (please see pagg.8-10).
b) We revised the short psychological testing description (please see pag. 8-11).

R1: The reference for the Italian translation and validation of the questionnaire should also be provided.

Authors: We specified that will be applied Italian validation and adaptation for all psychological tests. Regarding to the CAQ test we inserted the

reference of the original version of questionnaire for Cardiac Anxiety and we reported that we will follow the scoring of Italian adaptation elaborated by 'La Cattolica Research Group'. For both, please see pag. 9.

R1: Is it not clear why the authors listed the measures of quality of life as behavioral rather than psychological

Authors: The Protocol study is based on multiple measurements distributed on 2 groups: medical and psychological testing.

The Medical Measurements paragraph distributed the exams into 4 subgroups: 1) Clinical parameters, 2) Impedance parameters, 3) Laboratory parameters and, 4) Diagnostic-instrumental parameters.

The Psychological Measurements paragraph distributed the test into 3 subgroups: 1) emotional indexes, 2) cognitive indexes and 3) behavioral indexes. The emotional indexes will be investigated by test measuring emotive states of the patients; the cognitive indexes will provide the measure of cognitive state of the old patients and will be applied in the recruitment of study sample (one of the exclusion criteria is the presence of neurological disorders, i.e: the cognitive decline of mild cognitive impairment) and then the behavioral measures will consent to measure the observed behaviors of the patients in the daily living. All of three indexes will compose the psychological parameters of the patients.

R1: p. 8 – line 38 Please specify the acronyms LDL (low-density lipoprotein)

Authors: We made the suggested change. Please see pag. 9

R1: p.11 – line 27-28: I suggest the authors to remove the term 'outcomes' and to clarify the follow-up points

Authors: We removed the term outcomes and inserted the term 'parameters'. Please see pag. 10

R1: Please clearly describe the recruitment and screening process.
p.12 – line 10: it is suggested to replace the subtitle 'study design' with 'procedures'

Authors: We made the suggested change. Please see pag. 10

R1: It is recommended to add sections: 'expected results' and 'conclusions' at the end of the manuscript

Authors: As suggested by the Editor, no need to provide both the sections in this kind of the paper.

R1: p.13 – line 10: please replace the word 'CDV' with 'CVD'
Authors: We made the suggested change. Please see pag. 12

R1: In addition:
I recommend the authors specify acronyms only once in the text and use them consistently. For example: cardiovascular disease (CVD)
Authors: We made the suggested change. Please see pag. 13

I suggest the authors standardize verbal time to the future throughout the text. I also recommend to uniform line spacing throughout the manuscript
Authors: We tried to standardize the verbal time to the future. We highlighted in gray color that throughout the paper. Thanks!

Reviewer: 2 (R2)
Dr. Karla Sampaio, Postgraduate Program in Pharmaceutical Sciences
Please also see attached document.

R2: Comments to the Author:
The study protocol submitted by Giacomo and colleagues to the BMJ intends to implement a multidimensional model treatment for patients with cardiovascular disease based on the allostatic load index (ALI). The study is interesting and worthy investigating. However, some points need clarification.
Introduction

R2: The background is well-anchored on literature evidence. However, considering the endpoints, what do the authors expect to find by the end of the study? What is their hypothesis?
Authors: Thanks for the points. Regarding to the expected results, as suggested by Editor, we cannot provide them at this time of the research. Regarding to the hypothesis, we added in the paper, please see pag. 7.

R2: Objectives
The specific aims towards the secondary endpoints seem too broad. Can the authors be more specific regarding it?
Authors: As above, we added the hypothesis: that could be useful to better draw the endpoints (please see pag. 7). Anyway, we will conduct an

observational study on interplay between medical and psychological indexes and how those could impact the health management of patients into pharmacological and non-pharmacological treatments. The finding will be basilar for next interventional research protocol addressed to apply the CVD patient profiling into integrated clinical treatments'.

R2: Methods
1. How did the authors define the sample size? Please present the statistical parameters used to define it and the supporting reference.
Authors: We elaborated the sample size as reported in the Protocol on the basis of the theoretical framework discussed in the Introduction paragraph, but we didn't execute specific calculation. Our Protocol study is an observational study based on clinical population and further intervention protocol study will foresee the sample size calculation.

R2: 2. In the inclusion criteria, the authors state that they will enroll Heart failure patients. However, most evidence presented in the introduction, regarding the relationship between allostatic load and cardiovascular disease, is among hypertensive patients. If the idea is to develop a preventive model based on the AL Index, why not include hypertensive patients before heart failure dysfunction is installed?
Authors: We decide do not repeat the several studies already conducted and discussed in the literature: we wanted to study chronic CVD patients into polipharmacological treatments. As reported by the reviewer ('The background is well-anchored on literature evidence') that represents the framework of the study. Our scope is to provide improved data for clinical practice.

R2: 3. Explain why there is no control arm in the study design.
Authors: We didn't insert the control arm because we wanted to analyze the management of the disease, and it cannot be possible with health population. Actually, our study is observational and it could provide the elements for a related interventional protocol.

R2: 4. Explain the rationale for excluding patients undergoing pharmacological treatment for psychiatric disorders, once a great part of AL is dependent on psychological factors. Can this introduce a bias in the study?
Authors: In the psychological field that criterion is fundamental. The

psychiatric pattern isn't the Allostatic loading profile. Psychiatric disorders could
represents a bias for study.

R2: 5. How will the authors control patients' pharmacological treatment for other conditions straightly related to CVD such as diabetes?
Authors: Co-morbidities of CVD will be evaluated carefully by medical staff and medical measurements will be basilar to recruit the patients.

R2: 6. Justify the inclusion of only two laboratory parameters, once in the introduction, other biochemical markers are used to compose the ALI.
Authors: The inclusion criteria have been choose privileging the behavioral, cognitive and emotional measurements. In our study we wanted to apply the biopsychosocial approach and not just biomedical one, largely dealt with in literature. In our protocol, we will detect the medical parameters in usual care and biomedical markers were chosen as target for CV pathologies.

R2: 7. In the outcomes section, the authors describe a study sequence of visits at baseline, day 6, 12, and 18 months. However, in figure 2, another timeline interval is stated. Please justify the different intervals and present evidence for supporting the choice of time interval and duration of the study.
Authors: We made the changing. Now the paragraph reflects correctly the timeline represented in figure 2. Please see pag. 11.

R2: 8. It isn't clear what will be measured by the end of the study. What are the patients' outcomes to be analyzed?
Authors: As reported in paragraph 'Integration of clinical and psychological measurements: Allostatic load index' all measures will be processed to have an index for clinical practice'. At the end of the study, combined (clinical, instrumental and psychological) parameters will provide the health/disease progression and the adherence to the medication, as well the new adverse events and clinical sequelae (death, heart failure, hospital admission).

R2: 9. Will statistical analysis be blinded conducted?
Authors: We confirm that. Please see pag. 13

R2: 10. Provided a more detailed description of statistical analysis, providing supporting evidence.
Authors: As reported above, we cannot make so extensive changing in the Protocol Study and following the journal policy. We hope the Reviewer could understand our position. Anyway, we took care careful of that suggestion and we will go on in the next study.

VERSION 2 - AUTHOR RESPONSE
